# Biodistribution of cisplatin revealed by imaging mass cytometry identifies extensive collagen binding in tumor and normal tissues

**DOI:** 10.1038/srep36641

**Published:** 2016-11-04

**Authors:** Qing Chang, Olga I. Ornatsky, Iram Siddiqui, Rita Straus, Vladimir I. Baranov, David W. Hedley

**Affiliations:** 1 Fluidigm Canada Inc., 1380 Rodick Road, Markham, Ontario L3R 4G5, Canada; 2 Department of Pathology, Hospital for Sick Children, 555 University Avenue, Ontario M5G 1X8, Canada; 3 Division of Medical Oncology and Hematology, Princess Margaret Cancer Centre, 610 University Avenue, Toronto, Ontario M5G 2M9, Canada

## Abstract

Imaging mass cytometry was used for direct visualization of platinum localization in tissue sections from tumor and normal tissues of cisplatin-treated mice bearing pancreas cancer patient-derived xenografts. This recently-developed technology enabled simultaneous detection of multiple markers to define cell lineage, DNA damage response, cell proliferation and functional state, providing a highly detailed view of drug incorporation in tumor and normal tissues at the cellular level. A striking and unanticipated finding was the extensive binding of platinum to collagen fibers in both tumor and normal mouse tissues. Time course experiments indicated the slow release of stroma-bound platinum, although it is currently unclear if released platinum retains biological activity. Imaging mass cytometry offers a unique window into the *in vivo* effects of platinum compounds, and it is likely that this can be extended into the clinic in order to optimize the use of this important class of agent.

The platinum-containing drugs are used extensively in the treatment of solid tumors^1^,^2^,^3^,^4^,^5^. Cisplatin and the related compounds carboplatin and oxaliplatin form stable adducts with DNA, leading to the formation of lethal strand breaks^6^. In contrast to other DNA-damaging agents, rapidly proliferating normal tissues including bone marrow and intestinal mucosa are relatively resistant to cisplatin, and renal tubule damage is the limiting toxicity in humans^7^.

Pancreatic cancers developing in individuals with germline mutations in the breast cancer-related genes BRCA1 and BRCA2 are unusually responsive to cisplatin^8^,^9^. Thus, assignment of treatment towards platinum-containing regimens in BRCA-positive pancreatic cancer patients is an important step towards individualized treatment for these highly lethal cancers. However, clinical benefit is limited due to the development of drug resistance. Resistance to cisplatin is multifactorial, and includes alterations in drug uptake, efflux and detoxification that limit the initial DNA binding; enhanced capacity to excise DNA adducts; and suppression of death effector pathways^10^,^11^,^12^,^13^,^14^.

Mass cytometry is a technique for the high dimensional analysis of single cells in suspension. Unlike conventional fluorescence assays, antibodies are conjugated to metal-chelating polymers that bind stable isotopes, and cells are detected using inductively coupled plasma mass spectrometry (ICP-MS). By avoiding the problem of fluorescence spectral overlap, and exploiting the large number of isotopes currently available, mass cytometry allows the simultaneous measurement of up to 40 protein markers. Imaging mass cytometry (IMC) is an emerging technology based on principles similar to mass cytometry^15^. Instead of ionizing single cells in suspension, IMC uses a pulsed UV laser to ablate tissue sections, and a stream of inert gas to transfer the plumes of particles generated by the laser to the detector. Computer-generated images of antibody-labeled sections provide anatomical detail comparable to light microscopy. Similar to immunohistochemistry, IMC is readily applied to formalin-fixed, paraffin-embedded (FFPE) sections, and lends itself well to anatomical pathology. Analysis of the generated high dimensional data enables a systems biology approach to regulatory processes in heterogeneous cell populations^16^.

Previous studies have used bulk assays to measure the amount of Pt in tissues obtained from patients following cisplatin treatment^17^, with little information available about the localization of cisplatin within the tumor microenvironment and in normal tissues. Mass cytometry is able to detect elements in the mass range 75–209, and we have previously described its use to measure the uptake of platinum-based drugs in cell suspensions from human tumor xenografts^18^. Here we used IMC to study the tissue distribution and quantitation of cisplatin in conjunction with multiple biomarkers in pancreatic cancer patient-derived xenografts. These studies revealed extensive binding of cisplatin to collagen fibers in the tumor stroma, as well as in normal tissues including kidney, intestine and skin.

## Results

### Characterization of patient-derived xenografts

**Figure 1:**
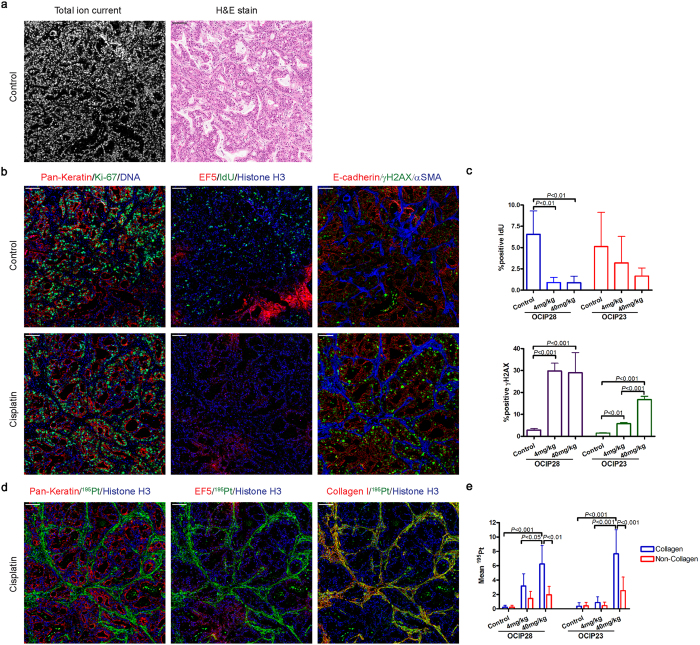
Cisplatin effects on tumor proliferation, DNA damage and cisplatin distribution in the tumor. **(a)** Representative total ion current image (left) and H&E stain (right) of control OCIP28. Scale bar = 100 μm. **(b)** Representative Pan-Keratin, Ki-67, DNA, EF5, IdU, Histone H3, E-cadherin, γH2AX, and αSMA images of tumors from control and cisplatin-treated (40 mg/kg for 24 h) mice. Scale bar = 100 μm. **(c)** Percentage of positive IdU (top) and γH2AX (bottom) bar graph of OCIP28 and OCIP23 xenografts. Quantitative results were obtained in all figures using the segmentation algorithm of Definiens Developer. **(d)** Representative Pan-Keratin, EF5, Collagen I, ^195^Pt, and Histone H3 images of cisplatin-treated (40 mg/kg for 24 h) in OCIP28. Scale bar = 100 μm. **(e)** Platinum distribution in collagen and non-collagen viable tumor tissue area in OCIP28 and OCIP23 xenografts.

Grayscale images of tissue sections generated from the total ion current (TIC) at the detector per pixel were used as a proxy to an optical image. TIC, as a computer-generated image, showed similar morphological features to hematoxylin/eosin (H&E) stained sections from the same tissue block. Although it is expected that the best analogy should be achieved with the greatest number of detected channels, rational selection of nuclei, membrane and stroma markers is helpful. As illustrated in Fig. 1 and Supplementary Fig. 1, the PDX models OCIP28 and OCIP23 have typical features of pancreatic ductal adenocarcinoma, with the malignant epithelium arranged into glandular structures embedded in a fibrous stroma. For illustrative purposes, combinations of images generated from the individual IMC markers were false colored and overlaid to show their spatial correlations. For example, as seen in Fig. 1b, cells incorporating IdU also express the proliferation marker Ki-67, and they are absent from regions of hypoxia marked by the tracer EF5, as we have previously observed by fluorescence imaging^19^. Expression of the DNA damage marker γH2AX is substantially confined to necrotic tissue adjacent to regions of hypoxia. Also seen are abundant cells expressing the mesenchymal marker αSMA, interspersed between the cytokeratin and E-cadherin-expressing epithelial cells. Following treatment with 4 mg/kg cisplatin, which roughly corresponds to the dose intensity used in humans, BRCA-mutant OCIP28 tumors showed loss of IdU incorporation and a striking increase in γH2AX expression, whereas these effects were much less apparent in BRCA-wild type OCIP23 that is resistant to cisplatin^20^. Even when the dose of cisplatin was increased to 40mg/kg the effects on IdU uptake and γH2AX in OCIP23 were less than those in the BRCA-mutant model at the lower dose, (Fig. 1c and Supplementary Fig. 1). Cell suspensions were prepared following published protocols^18^ from fresh tumor pieces after cisplatin treatment, and whole-cell analyses were made by mass cytometry (CyTOF^®^ 2). The values for Pt atoms/cell and IdU incorporation (iodine-^127^I/cell/minute) are shown in Supplementary Fig. 2, and are very similar to those previously obtained with cell line-derived xenografts^18^.

### Cisplatin distribution in tumor tissue

Images of each of the major isotopes of platinum were readily captured by IMC: the most abundant form, ^195^Pt, is used for illustration. Tissue sections prepared by formalin fixation and paraffin embedding undergo extensive washing and solvent treatment, which might remove loosely bound cisplatin. Therefore, we first examined paraffin-embedded and cryostat sections cut from snap frozen samples prepared from the same tumor. We found that the intensities and distribution patterns of platinum labeling were very similar, with no appreciable loss of signal in the paraffin sections, indicating that Pt remaining 24 hours after cisplatin treatment is, most likely, tissue-bound (Supplementary Fig. 3). Therefore, most of the work was done using FFPE sections. At 4 mg/kg cisplatin, Pt was detected above background in OCIP28 tumors, but not in OCIP23 where it was, however, readily detected at the higher dose. Unexpectedly, the intensity of ^195^Pt was considerably greater in the stromal component than in the tumor epithelial tissue. Additional staining for Collagen I showed co-localization with ^195^Pt, and it was intimately associated with the mesenchymal marker αSMA, as expected (Fig. 1d). Strong collagen binding of Pt was observed in both PDX models tested, and the intensity was highly significant when compared to epithelial tumor tissue (Fig. 1e).

### Cisplatin distribution in normal intestine

**Figure 2:**
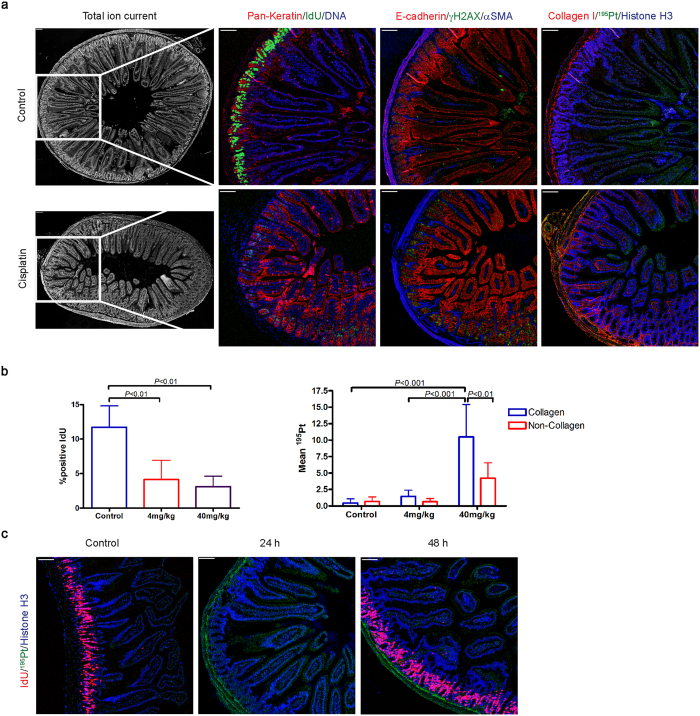
Cisplatin effects and distribution in the small intestine. **(a)** Representative total ion current images, Pan-Keratin, IdU, DNA, E-cadherin, γH2AX, and αSMA, Collagen I, ^195^Pt, and Histone H3 images of small intestine from control and cisplatin-treated (4 mg/kg for 24 h) OCIP28 PDX mice. Scale bar = 100 μm. **(b)** Percentage of positive IdU bar graph (left) and platinum distribution in collagen and non-collagen epithelial tissue area of small intestine. **(c)** Representative IdU, ^195^Pt, and Histone H3 images of control, cisplatin-treated (4 mg/kg for 24 h and 48 h) small intestine. Scale bar = 100 μm.

The small intestine and colon were harvested from mice receiving 4 mg/kg cisplatin, with IdU given 30 min prior to sacrifice. Paraffin sections were cut for IMC staining using the same antibody cocktail as for the tumor tissue (Table 1). As shown in Fig. 2, there was intense IdU uptake by the basal crypt cells of untreated control mice, whereas the labeling of colonic mucosa was lower (Supplementary Fig. 4). DNA synthesis was strongly inhibited following cisplatin treatment, accompanied by increased γH2AX labeling. As in tumor tissue, there was extensive binding of platinum to collagen in the lamina propria and outer wall of the small intestine, as well as in apical villous epithelial cells, and a similar pattern was also observed in the colon (Supplementary Fig. 4). Interestingly, at 48 hours after cisplatin treatment the IdU uptake by the basal crypt cells recovered, despite the persistence of platinum binding to collagen in the underlying basement membrane (Fig. 2c).

### Cisplatin distribution in skin

**Figure 3:**
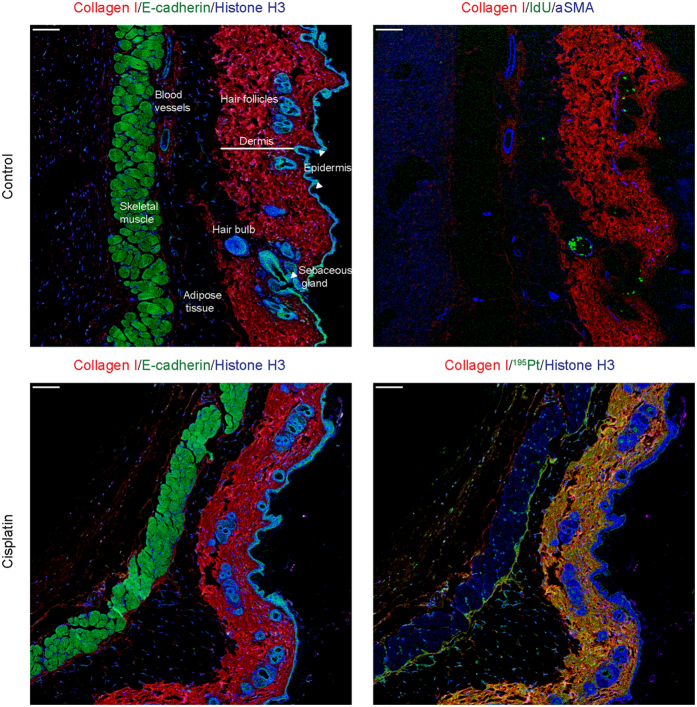
Histological features of skin identified by IMC, and distribution of platinum following treatment with cisplatin. Representative Collagen I, E-cadherin, Histone H3, IdU, and αSMA images of control (top) and cisplatin-treated (4 mg/kg for 24 h, bottom) mouse skin. Scale bar = 100 μm.

Sections cut through the thickness of the abdominal wall illustrate the histological features of several distinct tissues as viewed by IMC (Fig. 3). Similar to the intestinal wall and tumor tissue, binding of platinum to the dermal collagen was striking. Skin samples were taken from mice at 4 h, 24 h, 48 h, and seven days after cisplatin treatment, in order to determine the rate of loss of collagen-bound platinum. As illustrated in Supplementary Fig. 5a, dermal platinum decreased over time following a single dose of 4 mg/kg cisplatin (Supplementary Fig. 5b).

### Cisplatin in normal kidney and liver

**Figure 4:**
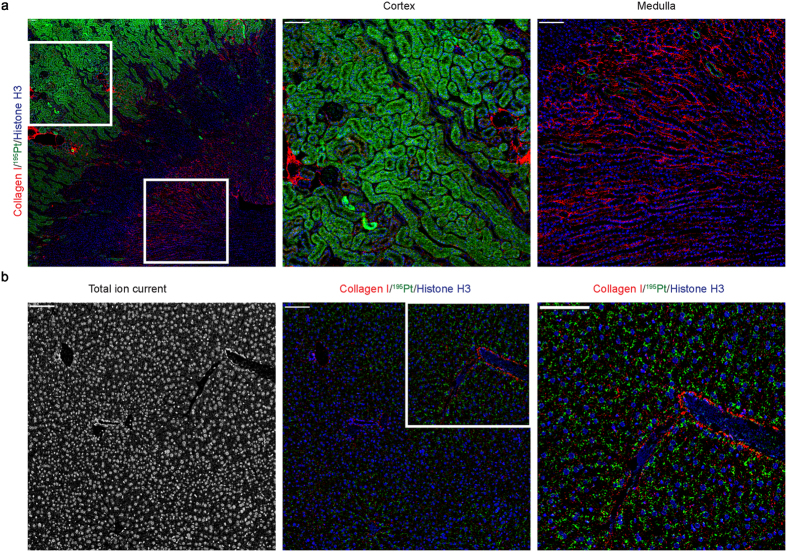
Cisplatin distribution in kidney and liver. **(a)** Representative Collagen I, ^195^Pt, and Histone H3 images of cisplatin-treated (4 mg/kg for 4 h) mouse kidney. Scale bar = 100 μm. **(b)** Representative total ion current image (left) and Collagen I, ^195^Pt, and Histone H3 image (right) of cisplatin-treated (4 mg/kg for 24 h) mouse liver.

The kidney and liver are the major organs involved in drug metabolism and excretion, although the liver plays a minor role in the case of cisplatin, which is mainly eliminated in the urine^21^. Renal tubular damage is the most serious toxic effect of cisplatin in humans^7^. Examination of the kidneys by IMC 24 hours after 4 mg/kg cisplatin administration showed a striking pattern, with large amounts of platinum in the cortical tubules and little accumulation in the glomeruli or renal medulla (Fig. 4a). Furthermore, high levels of platinum persisted for up to seven days post treatment, consistent with nephrotoxicity of this agent (Supplementary Fig. 6). In contrast, very little platinum was detected in the liver post-cisplatin (Fig. 4b). However, a distinct pattern of platinum accumulation was noted demonstrating a zone 3 sinusoidal and perivenular distribution that does not correlate with collagen (Fig. 4b, inset).

## Discussion

In the present paper, we exploited the unique potential of IMC to image the cellular content of platinum in BRCA-mutant and wild-type patient-derived pancreatic cancer xenograft-bearing mice treated with cisplatin, and in key normal host tissues. In combination with incorporation of ^127^I-IdU and the 2-nitroimidazole hypoxia tracer EF5, the expression of the DNA damage response marker γH2AX, and cell lineage-specific biomarkers, a number of novel and clinically important observations were made.

Platinum was readily detected in tissue sections following treatment with 4 mg/kg cisplatin, which gives drug exposure similar to that achieved in patients^22^. Although the free drug is rapidly cleared through the kidneys, it is extensively protein bound^21^,^23^ which is consistent with our observation that Pt levels in formalin-fixed, paraffin-embedded tissue are comparable to those in frozen sections. In the BRCA2-mutant PDX OCIP28, there was pronounced loss of IdU incorporation and increased γH2AX labeling following treatment with 4 mg/kg cisplatin, in line with our previous observation that this model is highly responsive to cisplatin^20^, and explained by deficient homologous repair (HR) consequent to the loss of BRCA2. In contrast, the HR-proficient model OCIP23 showed little response to 4 mg/kg cisplatin, and relatively modest effects on IdU and γH2AX when the dose was increased to 40 mg/kg. Although these findings are consistent with the clinical observation that pancreatic cancers developing in individuals with germline BRCA1/2 mutations can be highly sensitive to platinum drugs^9^, we note that Pt levels in the epithelial compartment of OCIP28 were significantly greater than those in OCIP23 following the clinically-relevant dose of 4 mg/kg. Resistance to cisplatin is multifactorial, and includes drug transport and detoxification pathways that limit cellular accumulation of the drug. It is possible that these mechanisms are expressed to a greater extent in OCIP23, and explain in part the differences in cisplatin sensitivity. This could be further studied exploiting the unique ability of IMC to measure Pt levels in combination with multiple antibodies directed towards proteins linked to cisplatin resistance, including the MRP2 efflux pump and enzymes involved in glutathione metabolism^11^,^12^,^13^, and extended to include additional PDX models. Fresh insights gained by such a study could have significant clinical utility, given the importance of platinum drugs in cancer treatment.

Using IMC, we obtained images of a range of normal tissues at a resolution comparable to that of light microscopy. Cisplatin is most active against proliferating cells, yet it appears to have little harmful effect on the intestinal mucosa of cancer patients. We were therefore interested to compare cisplatin accumulation and pharmacological effects in the small intestine basal crypt cells to those in the PDX. As shown in Fig. 2, IdU uptake by the crypt cells was greatly reduced 24 h following cisplatin, accompanied by increased γH2AX. This indicates that cisplatin has a significant acute effect on the small intestinal mucosa, but after 48 h IdU incorporation restarted, suggesting that these cells are able to repair drug-induced damage during a brief period of cell cycle arrest, and then resume normal growth.

Renal tubule damage is the most important side effect of cisplatin in humans, and it is only partially prevented by aggressive hydration regimens^7^. Accordingly, we noted intense Pt uptake by the tubular cells of the renal cortex that persisted over time, compared to the glomeruli and renal medulla, where Pt was virtually absent. This is in contrast with an earlier autopsy study of cancer patients receiving cisplatin, where the levels of Pt in cortex and medulla were similar^24^. This might be explained by species differences, or the longer duration of treatment in the patients. We suggest that the ability to image Pt at subcellular resolution using IMC offers a novel approach to the study of platinum-induced nephrotoxicity, and to explore new approaches to mitigate what remains a significant problem in clinical oncology.

Hepatotoxicity of platinum-based drugs has been previously described and well recognized^25^. Our observation that cisplatin accumulates in the liver, revealing a zone 3 sinusoidal and perivenular distribution, is of interest, since the mechanism of injury is believed to be damage to the sinusoidal endothelial cells in the liver followed by subsinusoidal fibrosis, obstruction and congestion, leading to veno-occlusive disease/sinusoidal obstruction syndrome^26^.

A striking and unanticipated finding was the extent of platinum binding to collagen, which was considerably greater than the epithelial cell Pt in both tumor tissue and normal intestine. We also noted intense Pt binding to dermal collagen; a site that is readily accessible to punch biopsy to allow a similar study in patients receiving cisplatin. A time course study found that dermal collagen-bound Pt is slowly released, but it is unclear if it remains pharmacologically active. However, it is of interest that platinum can be detected in patients’ plasma for several years post treatment with cisplatin, and that the levels are correlated with long term neuro- and ototoxicity^27^. Furthermore, ultrafiltrates of patient plasma were able to form DNA adducts, indicating the retention of biological activity^28^. The source of plasma platinum in these patients remains uncertain, although it is believed to be tissue-bound^28^. Early studies suggested that cisplatin is inactivated following protein binding^29^, but there are reports of benefit from intralesional treatment with cisplatin/collagen gels, with pharmacokinetic data consistent with slow release of drug in cancer patients^30^. Based on our IMC finding that cisplatin is extensively bound to collagen, we suggest this could have a significant effect within the tumor microenvironment due to the slow release of drug in sufficient amounts to affect tumor growth. It is reported that continuous exposure to low dose metronomic chemotherapy improves the therapeutic index of some cytotoxic agents^31^, although we are unaware that this has been tested with platinum compounds.

**Figure 5:**
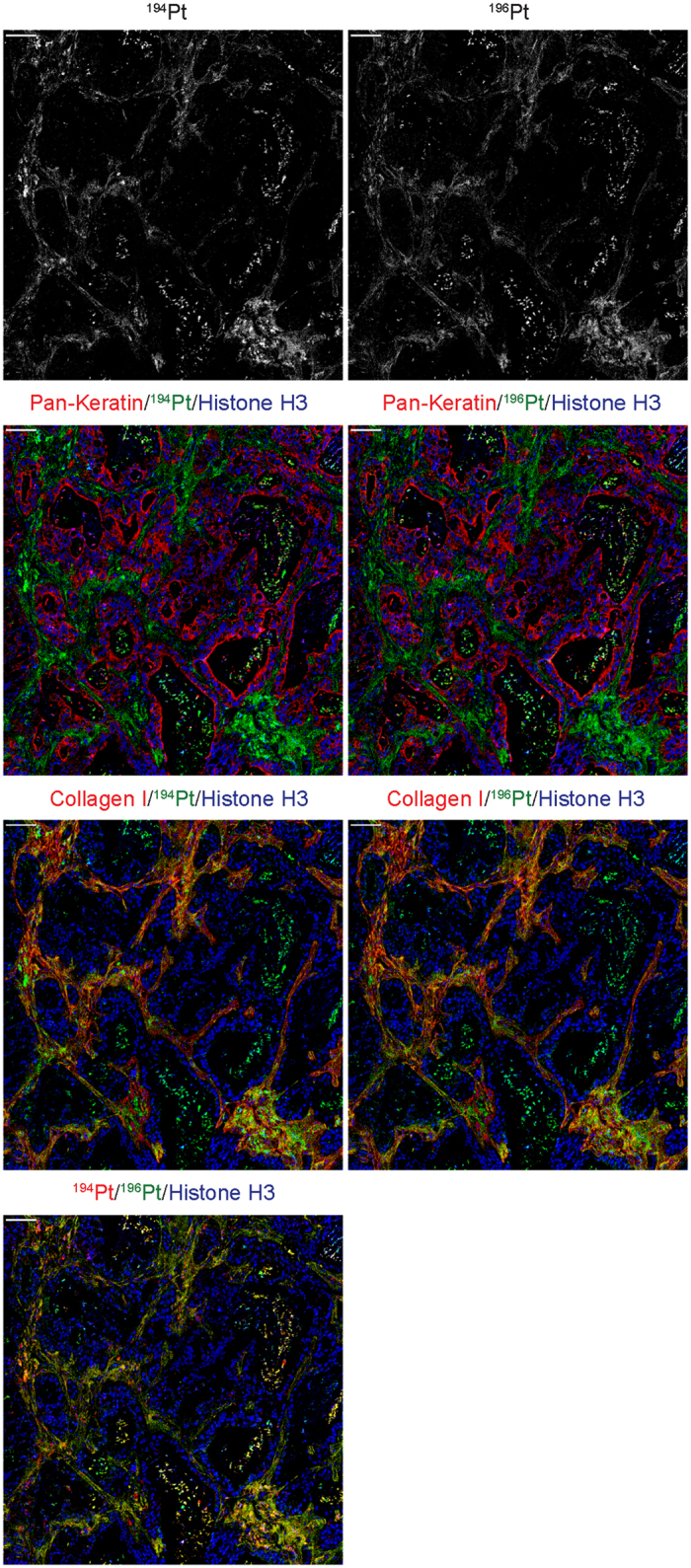
Kinetics of cisplatin distribution measured using monoisotopic cisplatin. Tumor-bearing mice were treated with ^194^Pt-cisplatin on Day 1, ^196^Pt-cisplatin on Day 5, and sacrificed on Day 6. The images are of a single tissue section showing the relative distribution and abundance of the two isotopes (top; grayscale), Pan-Keratin, Collagen I and Histone H3 vs Pt (lower panels). The bottom panel shows the two isotopes, with the color intensity indicating the preponderance of ^196^Pt at this time point. Scale bar = 100 μm.

The most significant new finding using IMC is the extensive binding of cisplatin to collagen within the tumor stroma and in normal tissues. The main limitations of the present study are: 1) it does not establish if collagen-bound cisplatin remains active; 2) the other clinically-important platinum drugs, carboplatin and oxaliplatin, were not included; 3) it does not establish if these effects also occur in cancer patients. To address this we used monoisotopic ^194^Pt and ^196^Pt forms of cisplatin for *in vivo* pulse/chase, to examine if collagen-bound cisplatin redistributes over time, and we show some preliminary data in Fig. 5. In this experiment, we treated tumor-bearing mice with 4 mg/kg ^194^Pt-cisplatin on Day 1, 4 mg/kg ^196^Pt-cisplatin on Day 5, and sacrificed on Day 6. As shown in Fig. 5, the two isotopes showed a similar distribution pattern, with cisplatin remaining substantially bound to collagen. However, we noted loss of ^194^Pt compared to ^196^Pt, indicating its slow dissociation from collagen.

Additionally, monoclonal antibodies to Pt-DNA adducts have been described^32^, and we will test if these can be used successfully to allow dual measurement of Pt biodistribution and drug-induced DNA damage using IMC. If so, this could be used to test if monoisotopic cisplatin, released over time from the tumor stroma, produces DNA damage in adjacent cancer cells. Although we have not yet applied IMC to carboplatin- or oxaliplatin- treated tumors, we have previously shown that Pt is readily detected in oxaliplatin-treated tumor cells using the CyTOF^18^, suggesting that IMC will be equally useful studying these agents in animal models, and also in patient samples.

Our plans for clinical translation involve two lines of research. One is a clinical study of collagen binding by cisplatin and oxaliplatin, based on IMC analysis of sequential skin biopsies obtained during treatment, compared to the corresponding plasma protein-bound and ultrafiltrable platinum. If collagen-bound platinum is the main source of plasma Pt post-treatment, then the two values are expected to correlate. In a separate group of patients treated with neoadjuvant platinum-based chemotherapy, we will determine the extent to which Pt binding to tumor collagen matches that in the patient’s skin, and ask if tumor Pt is correlated with treatment response. Our other research direction, and the original motivation for this work, is to investigate the mechanisms of platinum sensitivity and resistance in pancreatic cancers. It exploits the unique capability of IMC for single cell Pt measurements in combination with large panels of antibodies directed to the known determinants of treatment response, including proteins involved in drug transport and detoxification, as well as initial DNA damage and damage responses^33^.

In summary, the findings reported in this paper affirm that imaging mass cytometry represents a powerful new platform for studying complex biological processes in intact tissue. Although the main impact is expected to be in the area of cancer pathology, our results point to as-yet under-explored applications to the study of normal tissues and non-malignant pathology.

## Materials and Methods

### Experimental Design

The study was designed to test the potential of imaging mass cytometry to provide novel insights into the actions of cisplatin in patient-derived pancreatic cancer xenografts, selected based on the presence or absence of germline BRCA mutations. The research plan exploits IMC’s unique ability for combined single cell measurement of Pt with panels of antibodies towards determinants of cell lineage, treatment response and tumor microenvironment, as well as direct measurement of ^127^I-IdU incorporation to assess DNA synthesis.

### Sample Preparation

Animal experiments were carried out using protocols approved by the University Health Network Animal Care Committee. Fresh pancreatectomy samples that were superfluous to diagnostic needs were obtained from the University Health Network Tumor Tissue Bank (Toronto, Canada) according to a protocol that was approved by the University Health Network Research Ethics Board. Patient-derived xenografts (PDX) were established at the orthotopic site of 4- to 5-week-old severe combined immunodeficiency mice as previously described^19^,^34^. Non-tumor bearing mice and two models, designated as OCIP23 and OCIP28 were used for these experiments. These models were selected from our extensive pancreatic PDX resource on the basis of similar growth characteristics and significantly different cisplatin sensitivity, with the BRCA2-mutant model OCIP28 being highly sensitive, while the BRCA-wild type OCIP23 is resistant^20^. Cisplatin was obtained from the Princess Margaret Hospital pharmacy (Toronto, Canada). Duplicate mice in each group were treated with a single intraperitoneal dose of 0, 4 mg/kg or 40 mg/kg cisplatin for 24 h, or at 4 mg/kg and sacrificed after 4 h, 24 h, 48 h or 7 days for non-tumor bearing mice. The 2-nitroimidazole hypoxia probe EF5^19^ (obtained from Dr. Cameron J. Koch, University of Pennsylvania) at a dose of 30 mg/kg, and the thymidine analogue IdU (ThermoFisher, US) at 60 mg/kg, were given 4 h intravenously and 30 min intraperitoneally prior to sacrifice, respectively. Tumors and normal tissues (kidney, liver, small intestine, colon and skin) were excised, fixed and processed for paraffin embedding.

Additionally, monoisotopic cisplatin prepared from the stable isotopes ^194^Pt and ^196^Pt was obtained from Fluidigm Corporation (South San Francisco, CA, catalogue #201194 and 201196), sterile filtered, and injected intraperitoneally into tumor-bearing mice at a dose of 4 mg/kg. The first dose consisted of ^194^Pt-cisplatin, and a second dose of ^196^Pt-cisplatin was given 4 days later. Pairs of mice were sacrificed 24 h after the ^196^Pt-cisplatin dose.

### Imaging Mass Cytometry Staining Procedure for Tissue Sections

FFPE sections were dewaxed and rehydrated as routine. Heat-induced epitope retrieval was conducted in a water bath at 95 °C in antigen retrieval buffer (R&D Systems Inc., Minneapolis, MN) for 10 min. After immediate cooling, the sections were blocked with 3% BSA in PBS for 45 min. Samples were incubated overnight at 4 °C with a final concentration of 5–10 μg/mL of metal-conjugated antibody cocktails diluted in PBS/0.5% BSA (Table 1). Samples were then washed with 2× PBS/0.1% Triton X-100 and 2× PBS and exposed to 25 μM Ir-Intercalator (Fluidigm) for 30 min at RT for nuclei staining. The samples were rinsed twice in distilled water and air-dried before IMC analysis.

### Imaging Mass Cytometry Data Acquisition

The immunostained and dried samples are inserted into a novel laser ablation chamber (evolved from that described by Giensen, *et al*.^15^) where the tissue is scanned by a pulsed deep UV laser focused to a 1 micrometer diameter spot size. Tissue from an ablation spot is totally vaporized on each laser shot and the plume is carried with high time-fidelity into the inductively coupled plasma ion source for simultaneous analysis by the mass cytometer (Helios^TM^, Fluidigm). The metal isotopes associated with each spot are simultaneously measured and indexed against the location of each spot. The cross contamination between spots is less than 2% yielding sharp contrast images. The tissue is scanned spot-by-spot along a raster scan line, and sequential scan lines ultimately yield an intensity map of the target proteins throughout the tissue or the region of interest.

### Imaging Mass Cytometry Image Visualization and Data Analysis

Data processing was performed using in-house-developed Wolfram Mathematica^®^ (V10.3) algorithms. The transient signal data were exported from the mass cytometer in text format. The file consists of push numbers (i.e., time) in rows and mass channels in columns, and the measured values are given as ion counts. Images of each mass channel were reconstructed by plotting the laser shot signals in the same order that they were recorded, line scan by line scan. Multi-dimensional RGB images were overlaid for desired channel combinations. Comprehensive tissue cell segmentation was performed using Definiens^®^ Developer XD platform for digital pathology (Definiens AG; Munich, Germany). The Definiens Developer platform is used to generate tissue-specific image segmentation utilizing pathologist-trained rulesets. The rulesets contain machine learning algorithms which are applied to extract features that distinguish individual cell type such as stroma and epithelium, among others. The morphological assessment of both tumor and non-tumor tissue was performed by an expert pathologist (IS). Epithelial and stromal cell regions were selected based on morphology, e.g. shape of cell, size of cell, nuclear and cytoplasmic features, location of cells, etc. Subsequently, specific antibodies were used to confirm appropriate labeling for epithelial and stromal cells, i.e. collagen I and αSMA antibodies for stromal, and Pan-keratin and E-Cadherin antibodies for epithelial regions (Supplementary Fig. 7). Table 1 contains parameters used for the segmentation algorithm. After a supervised cell and tissue identification, a high-throughput quantification of cellular staining intensity provides multiple channel data quantification at the same time in one particular tissue area.

### Normalization and Error Propagation of Mean Values

The ^134^Xe^+^ ion, which is always present due to impurities in the argon plasma source in naturally occurring quantities, was monitored and used to normalize mean values for all reported channels for sensitivity fluctuations. ^134^Xe^+^ signal is uniformly distributed across all images, and its variability from image to image reflects a given state of the instrument tuning. The normalization algorithm includes construction of the ^134^Xe^+^ signal intensity histograms per image and following fit with a Gaussian function reflecting the counting statistics. The mean ^134^Xe^+^ signal values were used as normalization values. One image was arbitrarily chosen as a reference image (i.e. normalization factor = 1), and normalization factors were defined as the ratio of normalization values of a particular image and the reference image. Actual normalization of all channels was performed after the segmentation procedure.

### Statistical Analysis

The standard deviation of normalized values is taking into account the standard deviation of the measured values and the standard deviation of ^134^Xe ion signal. The means reported in the bar graphs are an average from multiple images. The standard deviations presented with these means account for additional errors in ion signal measurement that arise from variation between **a**. different areas of the same sample, and **b**. between different samples (i.e. different mice). Both errors arise from distinguishable sources, and the error bars represent the propagation of both errors. One-way ANOVA and Student’s t-test were used to determine statistical significance. All statistical analysis was done using GraphPad^TM^ Prism^®^ software (La Jolla, CA).

## Additional Information

**How to cite this article**: Chang, Q. *et al*. Biodistribution of cisplatin revealed by imaging mass cytometry identifies extensive collagen binding in tumor and normal tissues. *Sci. Rep.* **6**, 36641; doi: 10.1038/srep36641 (2016).

**Publisher’s note:** Springer Nature remains neutral with regard to jurisdictional claims in published maps and institutional affiliations.

## Supplementary Material

Supplementary Information

## Figures and Tables

**Table 1:** Metal-conjugated antibodies and element-containing reagents used to stain tissue sections for imaging mass cytometry.

Antibody/Reagent	Antibody Clone	Metal	Catalog number	Final concentration (μg/mL)
γH2AX	JBW301	^147^Sm	3147016A	5
p53	DO-7	^150^Nd	3150024A	5
Vimentin	RV202	^156^Gd	3156023A	5
*E-cadherin	24E10	^158^Gd	3158021A	5
EF5	ELK3-51	^159^Tb		10
VEGF	23410	^161^Dy	3161002B	5
*Pan-Keratin	C11	^162^Dy	3162027A	5
CK7	RCK105	^164^Dy	3164020A	5
β-catenin	D13A1	^165^Ho	3165027A	5
Ki-67	B56	^168^Er	3168007B	5
#Collagen I	Goat poly	^169^Tm		5
BRCA1	MS110	^172^Yb		5
#αSMA	1A4	^175^Lu		5
Histone H3	D1H2	^176^Yb	3176016A	5
*Intercalator		^191^Ir, ^193^Ir	201192B	25 µM
IdU		^127^I		60 mg/kg

^*^Denotes parameters used in cell segmentation by the Definiens Developer software.

^#^Denotes parameters used to identify collagen and non-collagen regions.
