# Supplementary material for: Biodistribution of cisplatin revealed by imaging mass cytometry identifies extensive collagen binding in tumor and normal tissues
Source: Sci Rep. 2016 Nov 4;6:36641. doi: 10.1038/srep36641 (PMC5095658; doi:10.1038/srep36641)
Supplement: Supplementary Information [file srep36641-s1.doc]

Biodistribution of cisplatin revealed by imaging mass cytometry identifies extensive collagen binding in tumor and normal tissues.

**Authors:** Qing Chang^1^, Olga I. Ornatsky^1^, Iram Siddiqui^2^, Rita Straus^1^, Vladimir I. Baranov^1^ and David W. Hedley^3,*^

**Affiliations:**

^1^Fluidigm Canada Inc., 1380 Rodick Road, Markham, Ontario L3R 4G5, Canada

^2^Department of Pathology, Hospital for Sick Children, 555 University Avenue, Ontario M5G 1X8, Canada

^3^Division of Medical Oncology and Hematology, Princess Margaret Cancer Centre, 610 University Avenue, Toronto, Ontario M5G 2M9, Canada

*To whom correspondence should be addressed: david.hedley@uhn.ca

Supplementary Materials:

Supplementary Methods:

Single cell suspensions analyzed by mass cytometry

Single cell suspensions from tumors were prepared by an enzymatic technique (Collagenase XI, Protease, and DNase I cocktail; Sigma, Mississauga, ON, Canada), filtered, washed and counted. Aliquots of single cell suspensions were fixed in 4% formaldehyde for 10 min followed by 0.1% TX-100 to optimize intracellular protein staining, and then analyzed immediately, or stored in 20% glycerol +10% FBS at -20°C. Mass cytometry was done using a Helios^TM^ system, and data analyzed using FlowJo Ver.10.1r5 (FlowJo LLC, Ashland, OR).

**Preparation of frozen sections**

Immediately after excision tumors were embedded in OCT compound (Tissue-Tek® Sakura® Finetek) and flash frozen in liquid nitrogen. Samples were then stored at -80 °C. Five micron thick cryostat sections were cut using a Shandon Crytotome (Thermo Fisher Scientific, Mississauga, ON, Canada).

Supplementary Figures:

Supplementary Figure 1. Cisplatin effects on tumor proliferation, DNA damage and ^195^Pt distribution in OCIP23.

Supplementary Figure. 2. Cisplatin effects on cell proliferation and platinum uptake determined by mass cytometry.

Supplementary Figure 3. Platinum distribution in cryostat and FFPE sections.

Supplementary Figure 4. Platinum distribution in large intestine.

Supplementary Figure 5. Platinum distribution in non-tumor bearing mouse skin.

Supplementary Figure 6. Platinum distribution in non-tumor bearing mouse kidney.

Supplementary Figure 7. Definiens Developer image analysis.


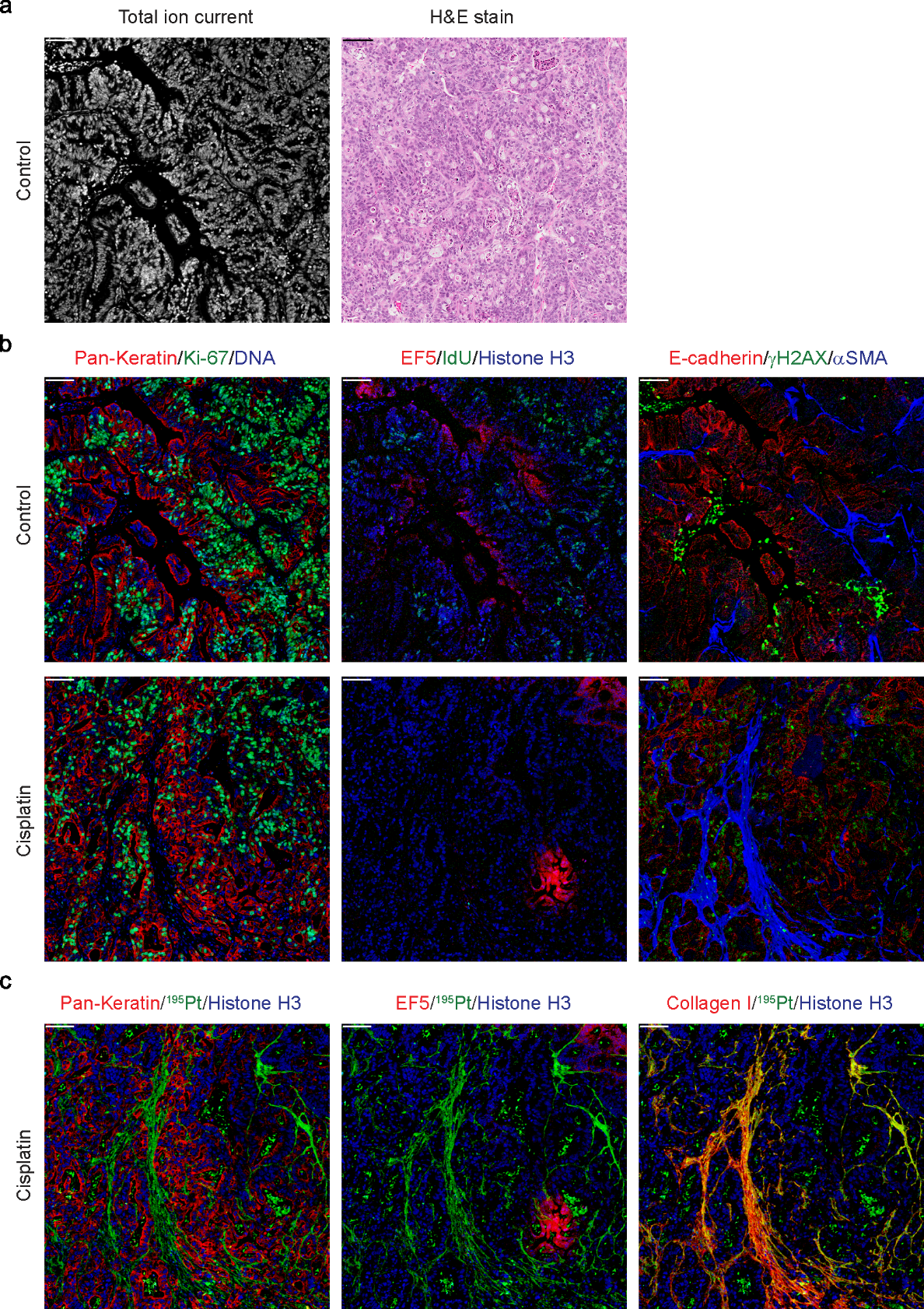


**Supplementary Figure 1** Cisplatin effects on tumor proliferation, DNA damage and ^195^Pt distribution in OCIP23. **(a)** Representative total ion current image (left) and H&E stain (right) of control OCIP23. Scale bar = 100 µm. **(b)** Representative Pan-Keratin, Ki-67, DNA, EF5, IdU, Histone H3, E-cadherin, γH2AX, and αSMA images of OCIP23, control and 40 mg/kg cisplatin-treated mice for 24 h. Scale bar = 100 µm. **(c)** Representative Pan-Keratin, EF5, Collagen I, ^195^Pt, and Histone H3 images of cisplatin-treated (40 mg/kg for 24 h) OCIP23 tumor-bearing mice. Scale bar = 100 µm.


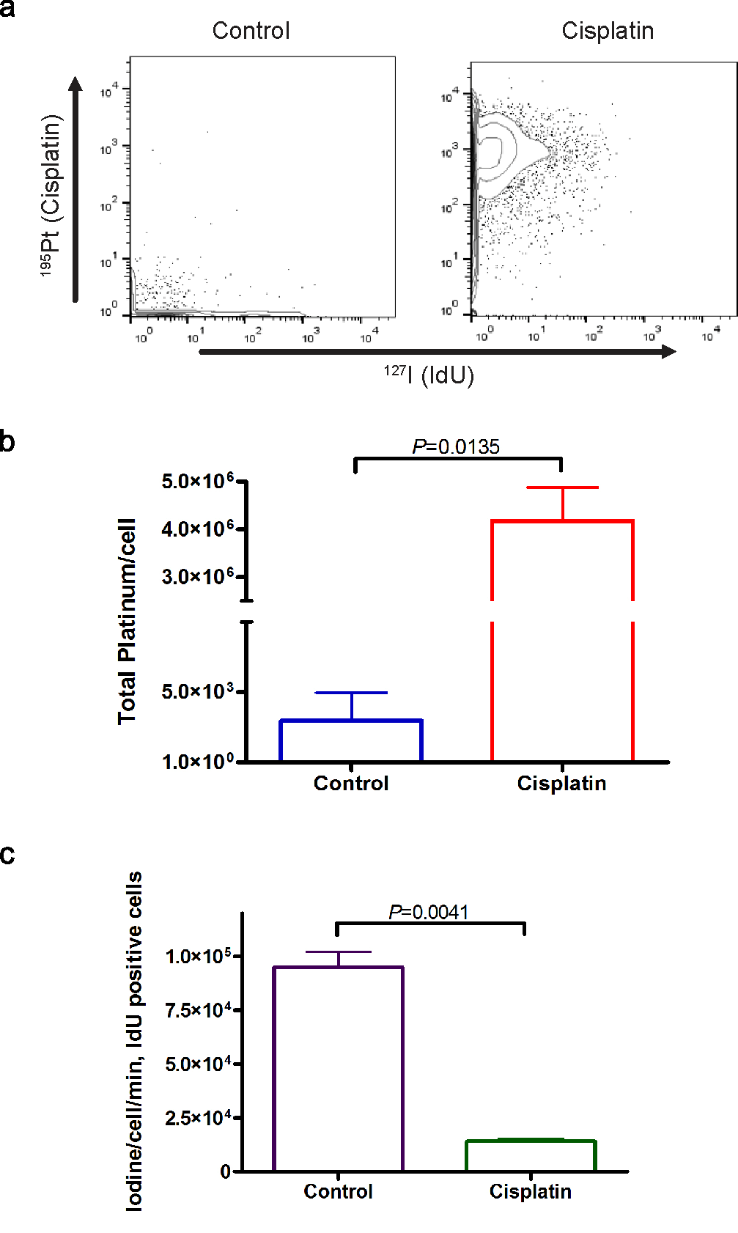


**Supplementary Figure 2** Cisplatin effects on cell proliferation and platinum uptake determined by mass cytometry. **(a)** Representative plots of ^127^I versus ^195^Pt in control and cisplatin-treated (40 mg/kg for 24 h) OCIP28 tumor-bearing mice. **(b)** Total platinum uptake of control and cisplatin-treated (40 mg/kg for 24 h) OCIP28 tumors. **(c)** Iodine atoms per cell data following a 30 min IdU pulse for identification of S-phase cells following a single dose of cisplatin.

**
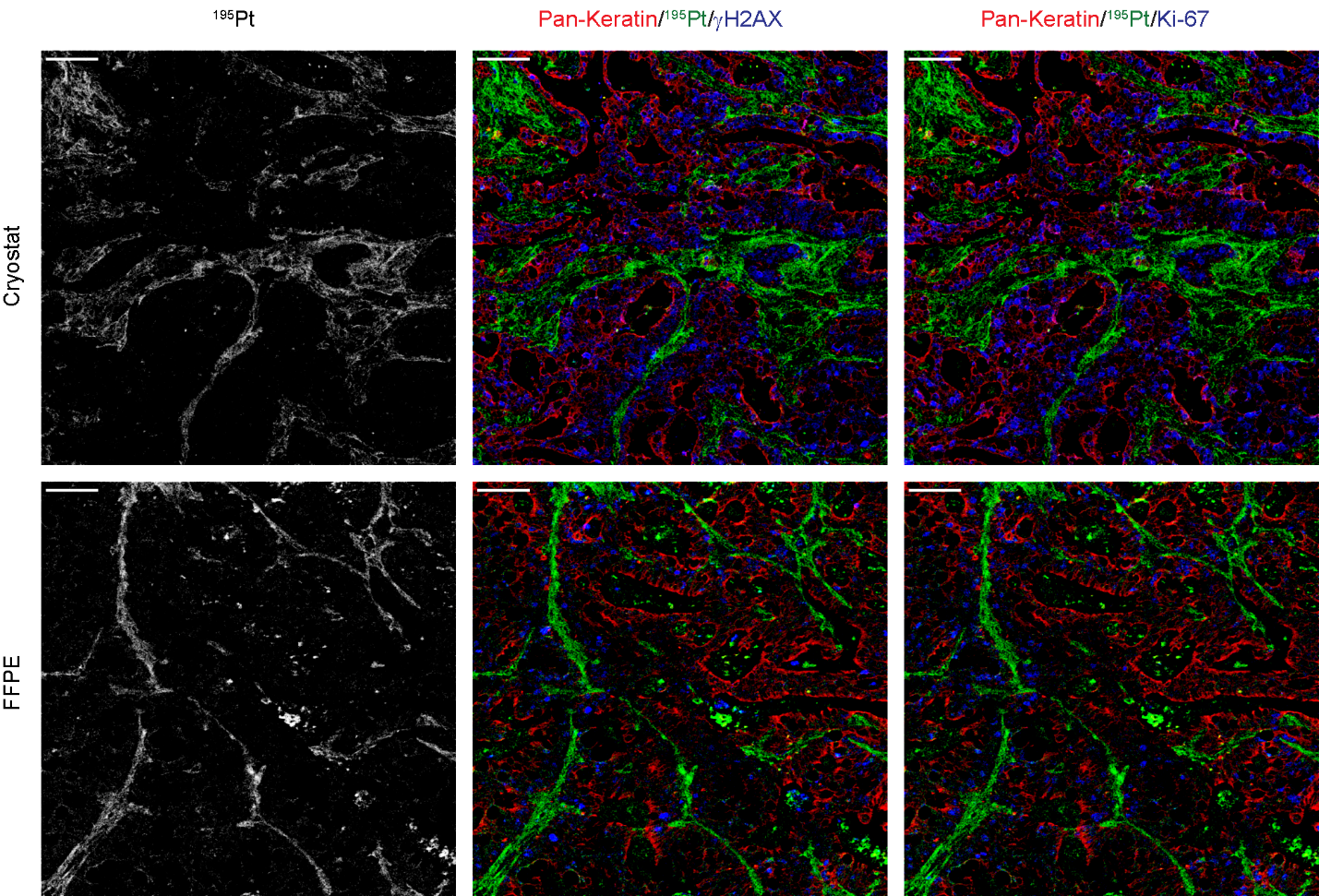
 Supplementary Figure 3** Platinum distribution in cryostat and FFPE sections. Representative ^195^Pt, Pan-Keratin, Ki-67, and γH2AX images of OCIP28 cisplatin-treated (40 mg/kg for 24 h) cryostat (top) and FFPE tumor sections (bottom). Scale bar = 100 µm.

**
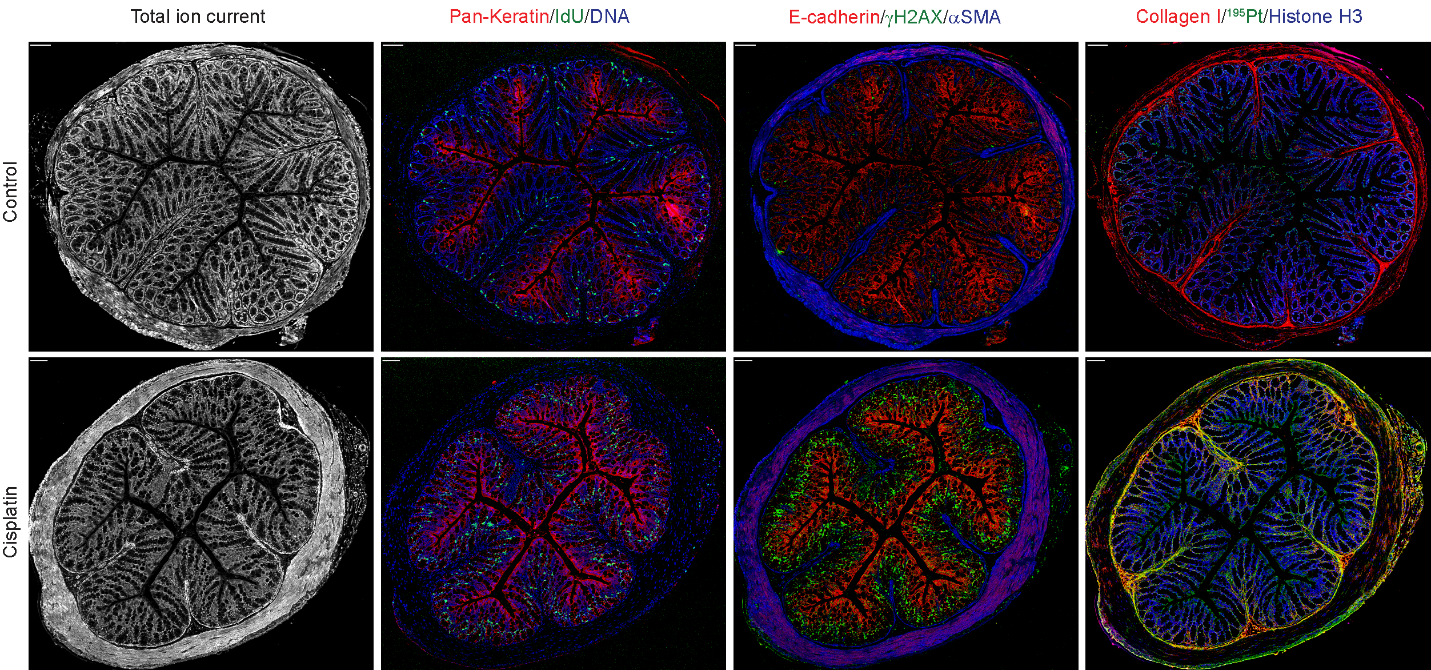
**

**Supplementary Figure 4** Platinum distribution in large intestine. Representative total ion current, Pan-Keratin, IdU, DNA, E-cadherin, γH2AX, αSMA, Collagen I, ^195^Pt, and Histone H3 images of large intestine from control (top) and cisplatin-treated (40 mg/kg for 24h, bottom) OCIP23 mice. Scale bar = 100 µm.

**
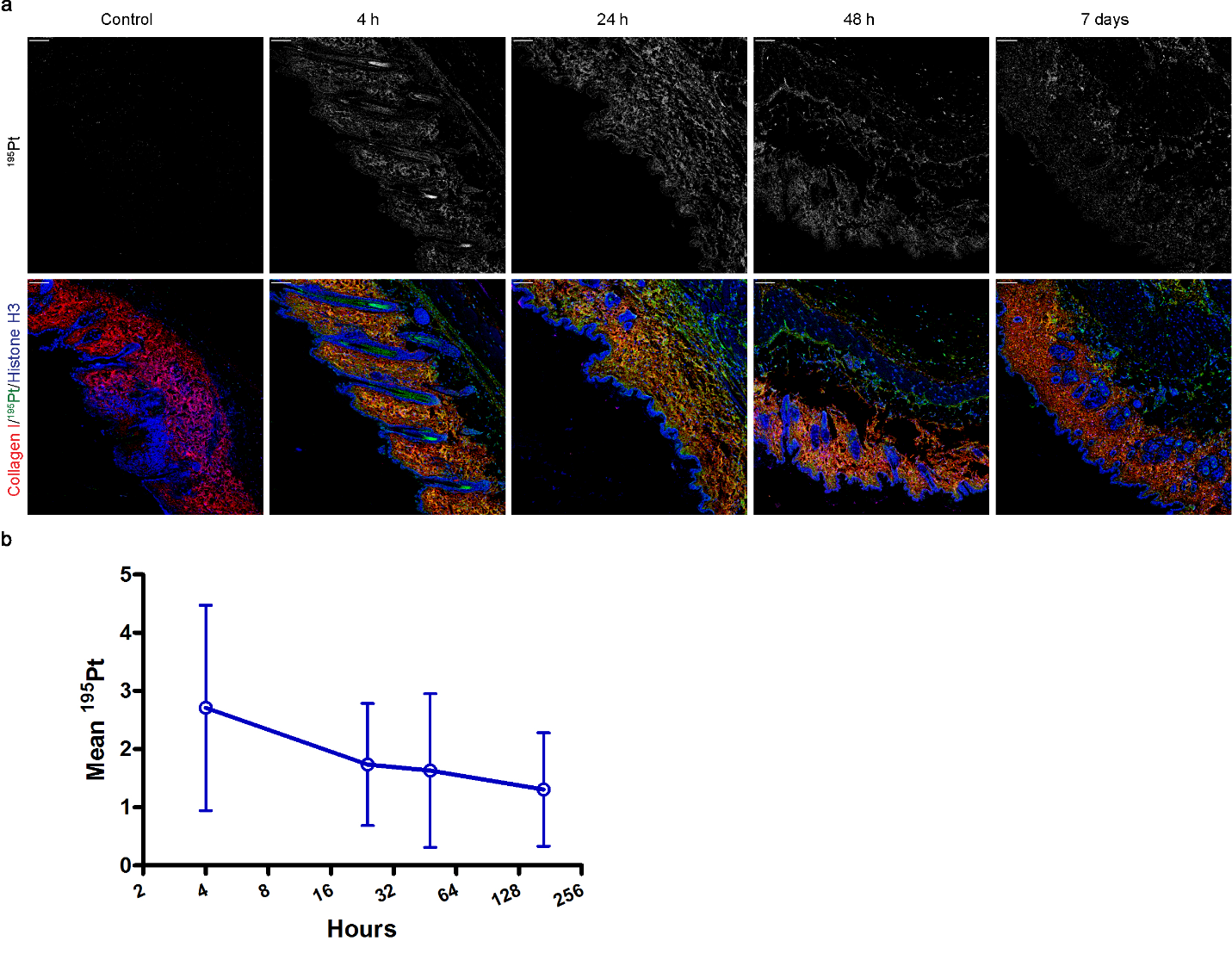
 Supplementary Figure 5** Platinum distribution in non-tumor bearing mouse skin. **(a)** Representative ^195^Pt (gray, top) and Collagen I, ^195^Pt, and Histone H3 (bottom) images of control and cisplatin-treated (4 mg/kg for 4 h, 24 h, 48 h, and 7 days) mouse skin. Scale bar = 100 µm. **(b)** Time course of platinum distribution in dermal collagen.

**
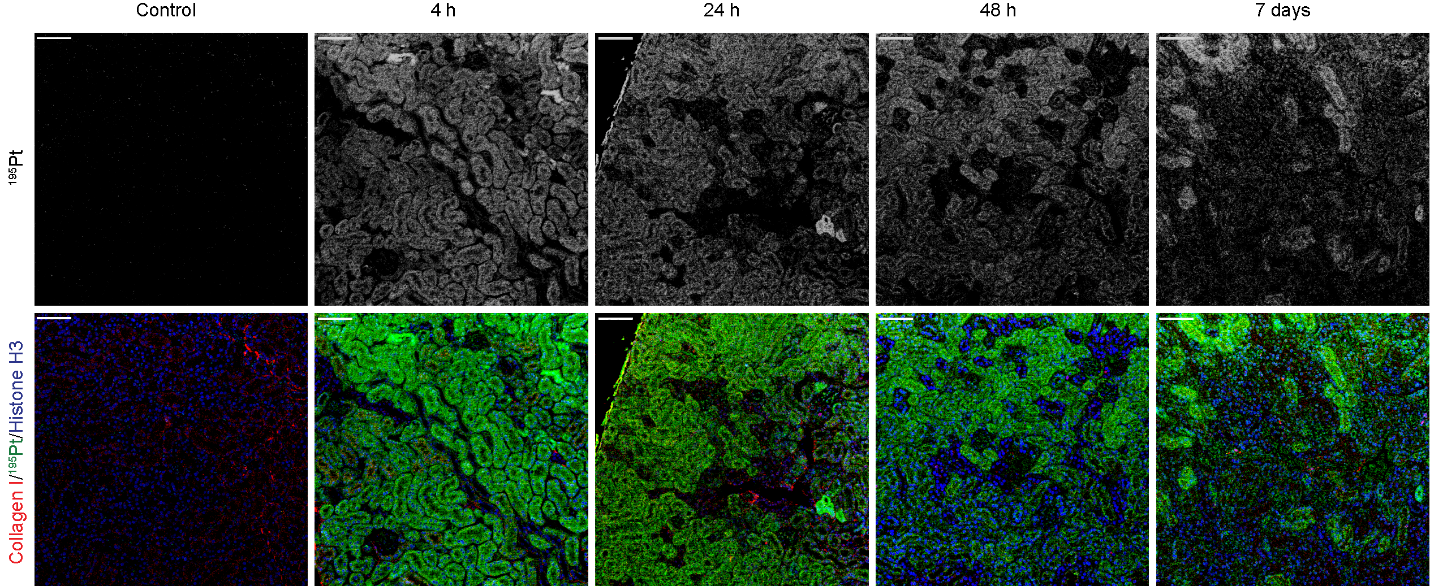
 Supplementary Figure 6** Platinum distribution in non-tumor bearing mouse kidney. Representative ^195^Pt (gray, top) and Collagen I, ^195^Pt, and Histone H3 (bottom) images of control and cisplatin-treated (4 mg/kg for 4 h, 24 h, 48 h, and 7 days) mouse kidney. Scale bar = 100 µm.

**
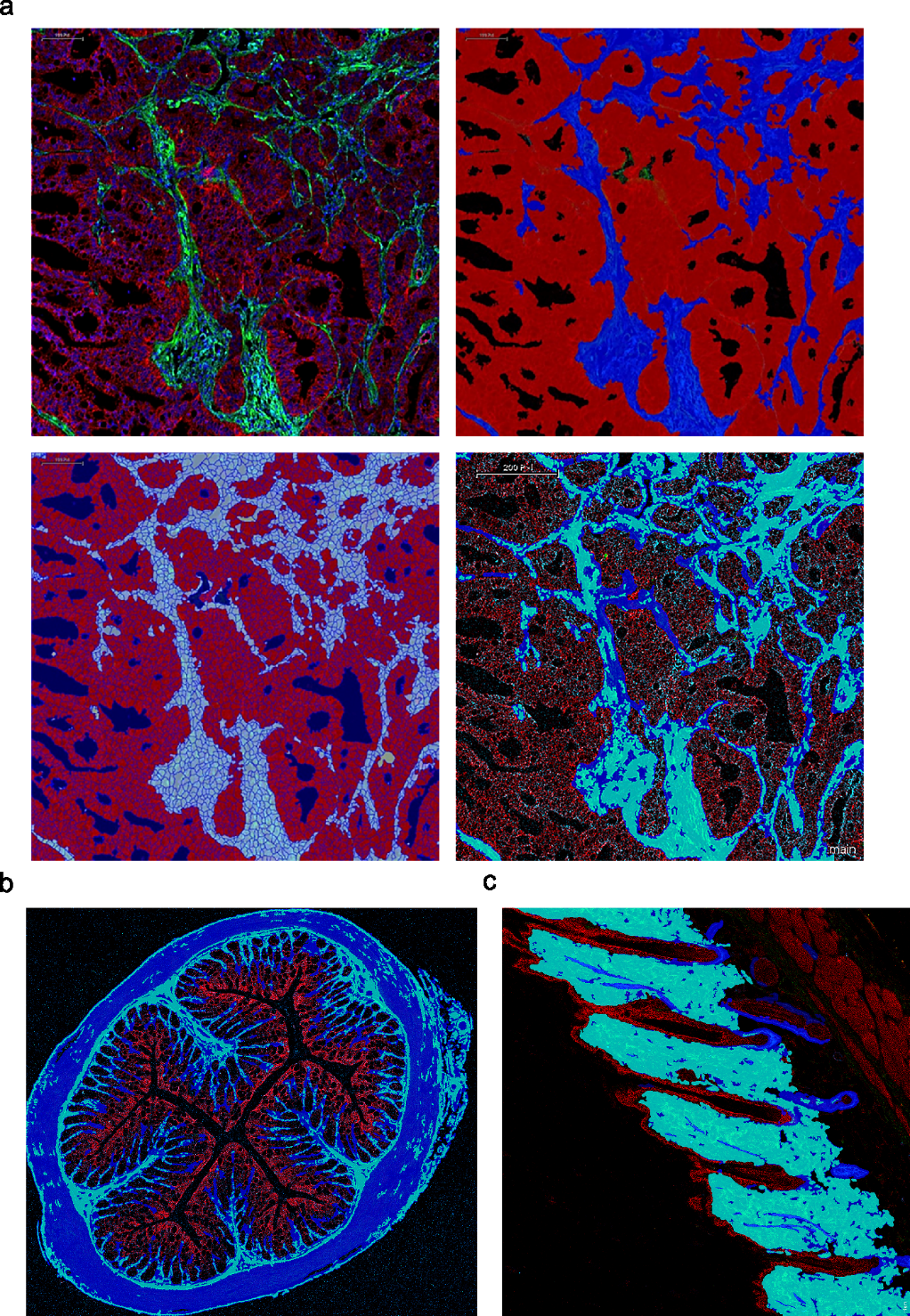
**

**Supplementary Figure 7** Definiens Developer image analysis. **(a)** Representative image of cisplatin-treated (40 mg/kg for 24 h) OCIP28 tumor. Top left: E-cadherin (red), γH2AX (green), DNA (blue), and Collagen I (cyan). Top right: the epithelium layer (red) is identified based on E-cadherin and Pan-Keratin. The stroma layer (green) is identified based on αSMA and Collagen I. Lumen is in black. Bottom left: Epithelial cells (red) and stromal cells (cyan) are identified based on DNA, and grown based on the ratios of stain present. Bottom right: The collagen layer (cyan) is identified from the stroma layer (blue). **(b)** The collagen layer (cyan) is identified from the stroma layer (blue) in mouse large intestine. **(c)** The collagen layer (cyan) is identified from the stroma layer (blue) in mouse skin.
